# Gut microbial-derived metabolites: key players in kidney disease and renal fibrosis

**DOI:** 10.7150/ijbs.133969

**Published:** 2026-05-18

**Authors:** Wan-Ying Zhang, Yu-Lu Zhang, Qing-Qing Yu, Jing-Teng Zhou, Hua Miao, Li-Min Liu, Ying-Yong Zhao

**Affiliations:** 1 School of Pharmaceutical Sciences, Zhejiang Chinese Medical University, No. 548 Binwen Road, Hangzhou, Zhejiang 310053, China.; 2 School of Pharmaceutical Sciences, The First Affiliated Hospital of Zhejiang Chinese Medical University, No. 54 Youdian Road, Hangzhou 310003, China.; 3 School of Medicine, Northwest University, No. 229 Taibai North Road, Xi'an, Shaanxi, 710069, China.; 4 State Key Laboratory of Kidney Diseases, First Medical Center of Chinese PLA General Hospital, No. 28 Fuxing Road, Beijing 100853, China.

**Keywords:** gut microbiota, acute kidney injury, chronic kidney disease, renal fibrosis, short-chain fatty acids, natural products

## Abstract

Kidney disease is a major global public health problem that affects 15-20% of adults globally. Increasing evidence shows that kidney injury results in intestinal barrier dysfunction, microbial dysbiosis, and microbial-derived metabolite disorder. Microbial-derived metabolites are recognized as multi-kingdom intermediates. The alterations of the gut microbiota lead to the reductions of short-chain fatty acids including acetate, butyrate, and propionate, while excessive accumulation of uremic toxins, including indoxyl sulfate, indole-3-acetic acid, trimethylamine-N-oxide, and p-cresyl sulphate that involved in renal disease. Various effects are moderated by regulating aryl hydrocarbon receptor, G-protein-coupled receptor 43, Toll-like receptor 4, domain-like receptor family protein 3, phosphatidylinositol-3 kinase, inhibitor of kappa B /nuclear factor kappa B and kelch-like ECH-associated protein 1/nuclear factor erythroid 2-related factor 2 pathways via gut microbiota-derived metabolites. Among them, tryptophan metabolites are considered pivotal in the communication between gut microbiota and kidneys. This review summarizes current understanding of the role of gut microbial-derived metabolites in acute kidney injury and chronic kidney disease including diabetic kidney disease, immunoglobulin A nephropathy and membranous nephropathy, explain the underlying pathophysiology of these associations and pinpoint potential targets for the future precision-based modulation of therapies. This review also explores therapeutic options, such as natural products, prebiotics, probiotics, and renal replacement therapy, for targeting gut microbiota dysbiosis and their metabolites in patients with kidney diseases to provide a more concept-driven and precise therapy strategy.

## 1. Introduction

Kidney disease is a major global health burden that affects more than 15-20% of the worldwide population 1, 2. Kidney disease including acute kidney injury (AKI) and chronic kidney disease (CKD) is characterized by a progressive decline in renal function accompanied by considerable morbidity and mortality 3, 4. Current interventions for renal disease, including renal transplantation and dialysis, are costly and not curative 5-7. Renal fibrosis, including renal tubulointerstitial fibrosis and glomerular sclerosis, is a chronic and progressive process 8. Renal fibrosis is characterized by massive activation and proliferation of myofibroblasts, macrophages and mesangial cells that lead to the excessive deposition of extracellular matrix and tissue scarring 9-13. Regardless of the underlying aetiology, is the ultimate common pathway for almost all types of chronic repetitive injury in the kidneys, ultimately leading to end-stage renal disease (ESRD) 9, 14. Substantial publications have dictated that renal fibrosis is extensively related to diverse signals such as hyperactive renin-angiotensin system, high mobility group box 1 and aryl hydrocarbon receptor (AHR) 15-17, and profibrotic pathways such as inhibitor of kappa B(IƙB)/nuclear factor kappa B (NF-ƙB), transforming growth factor β1 (TGF-β1)/suppressor of mothers against decapentaplegic (Smad) and Wnt1/β-catenin 18, 19. Moreover, emerging multi-omics (genomics, epigenetics, transcriptomics, proteomics, metabolomics, lipidomics and phenomics)-based molecular mechanisms highlighted that renal fibrosis is associated with gut microbial dysbiosis 20, 21, non-coding ribonucleic acids (ncRNAs) dysregulation 21-24 and altered levels in metabolites including tryptophan and lipid (phosphatidylcholines and bile acids) metabolisms 25-29.

Ample evidence has shown that renal fibrosis leads to intestinal epithelial cell barrier injury that increases intestinal permeability and gut microbiota dysbiosis that causes altered microbial-derived metabolites, such as indoxyl sulfate (IS), trimethylamine-N-oxide (TMAO), p-cresyl sulphate (PCS), indole-3-acetic acid (IAA), hyodeoxycholic acid and 1,5-anhydroglucitol, that correlate with renal functions 30, 31. These metabolites leak into bloodstream through damaged intestinal epithelial cell barrier, and mediate systemic and local low-level inflammation that further aggravates kidney injury and exacerbates progressive renal fibrosis by regulating a variety of signalling pathways, such as AHR, farnesoid X receptor, glucagon-like peptide-1 receptor (GLP-1), and transmembrane G protein-coupled bile acid receptor 5 32-34. Recently, integrating metagenomics and metabolomics reveals that renal fibrosis is related to gut microbial dysbiosis that leads to the aberrant microbial-derived metabolites 35, 36. Oxidative stress and inflammation play a central role in the progression and outcome of renal disease 37. Studies have revealed that CKD results in intestinal inflammation and impaired epithelial barrier function, which leads to an accelerated systemic translocation of bacteria-derived uremic toxins 38. Moreover, the aberrant gut microbiota in CKD patients results in metabolite disorder and ultimately exacerbates clinical outcomes 36, 39-41. Therefore, modulating gut-metabolite-kidney axis could be an effective approach to disease intervention.

This review summarizes the recent insights into the roles of gut microbiota-derived metabolites, such as short-chain fatty acids (SCFAs), microbial-derived tryptophan metabolites including IS, IAA, indole-3-propionic acid (IPA) and indole-3-aldehyde (IAld), TMAO, PCS and polyamines in health and kidney disease. The focus is on the regulatory functions of microbial metabolites in kidney disease, and their potential role in facilitating communication between gut and kidneys is explored. We also discuss current therapeutic strategies targeting the modulation of gut microbiota-derived metabolites through dialysis (hemodialysis, peritoneal dialysis and colonic dialysis), kidney transplantation (KT), natural product interventions, microecological therapies (probiotics, prebiotics and synbiotics), and dietary interventions in the management of kidney disease.

## 2. Gut microbiota and microbial-derived metabolites in health

### 2.1. Gut microbiota in health

**Figure 1:**
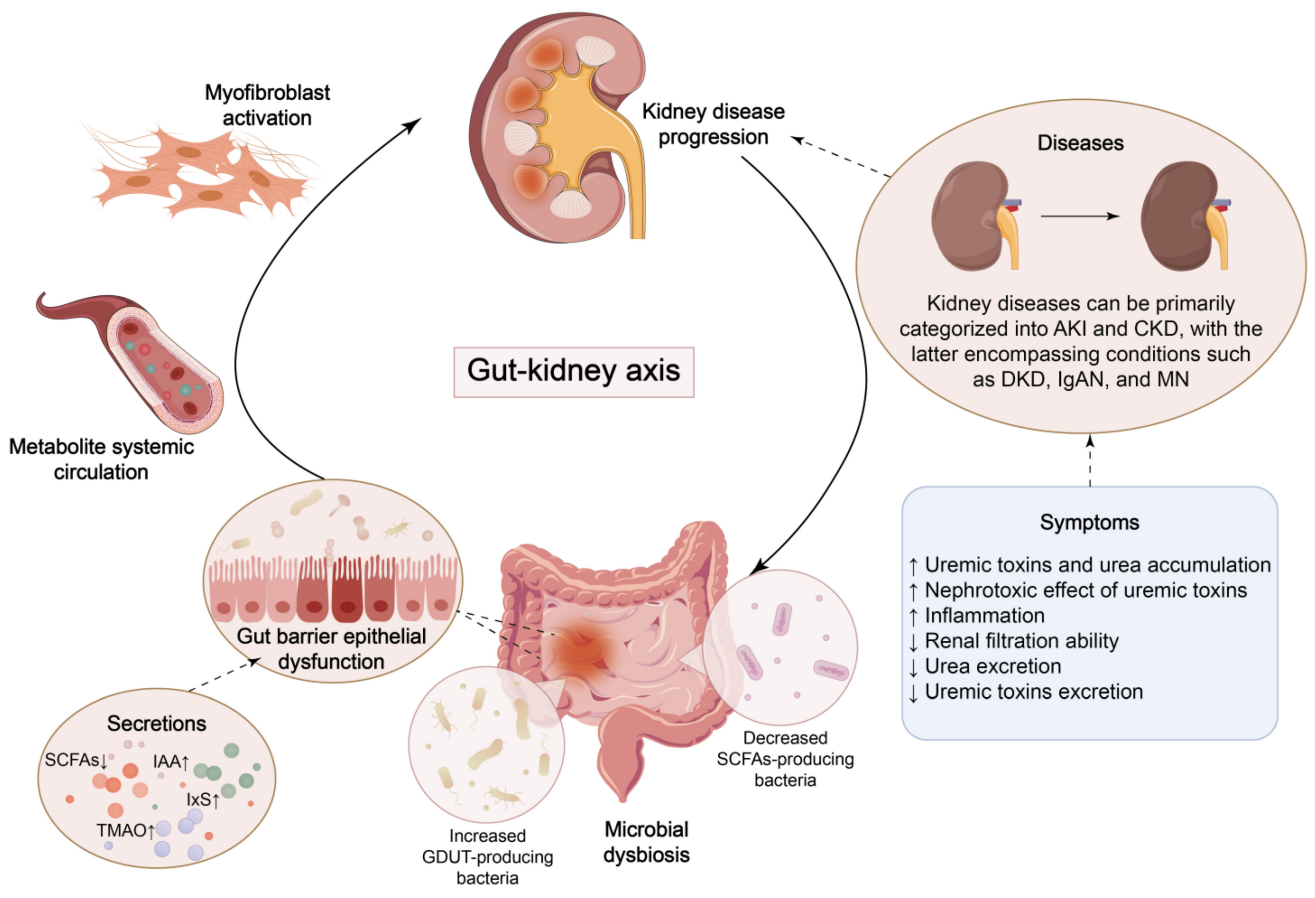
** Crosstalk between kidney disease and microbial dysbiosis.** Dysbiosis of gut bacteria leads to the accumulation of uremic toxins and impairment of the gut barrier, which triggers renal inflammation and fibrosis. The progression of kidney disease, in turn, exacerbates gut dysfunction.

Over the past decade, research has demonstrated that the extensive community of micro-organisms residing in the gut, known as the gut microbiota, is closely associated with human health and disease, partly due to its impact on systemic immune responses 42, 43. Gut microbiota can synthesize multiple vitamins and essential amino acids necessary for human development and growth, promote intestinal peristalsis, and maintain intestinal function 44. Four dominant phyla, Firmicutes, Bacteroidetes, Actinobacteria, and Verrucomicrobia, account for 99% of healthy human gut bacteria, with Firmicutes and Bacteroidetes dominating at a combined proportion of around 90% 45-47. Under normal circumstances, the gut microbiota maintains a symbiotic relationship with the host, which contribute to the therapy of metabolic dysfunction in humans, influencing intestinal immunity, tolerance, and sensitivity to inflammation 48. The theory of the gut-kidney axis has arisen collectively, highlighting the bidirectional communication between renal function and gut microbiota (Figure 1).

### 2.2. Gut microbiota and microbial-derived metabolites in health

#### 2.2.1. SCFAs

**Figure 2:**
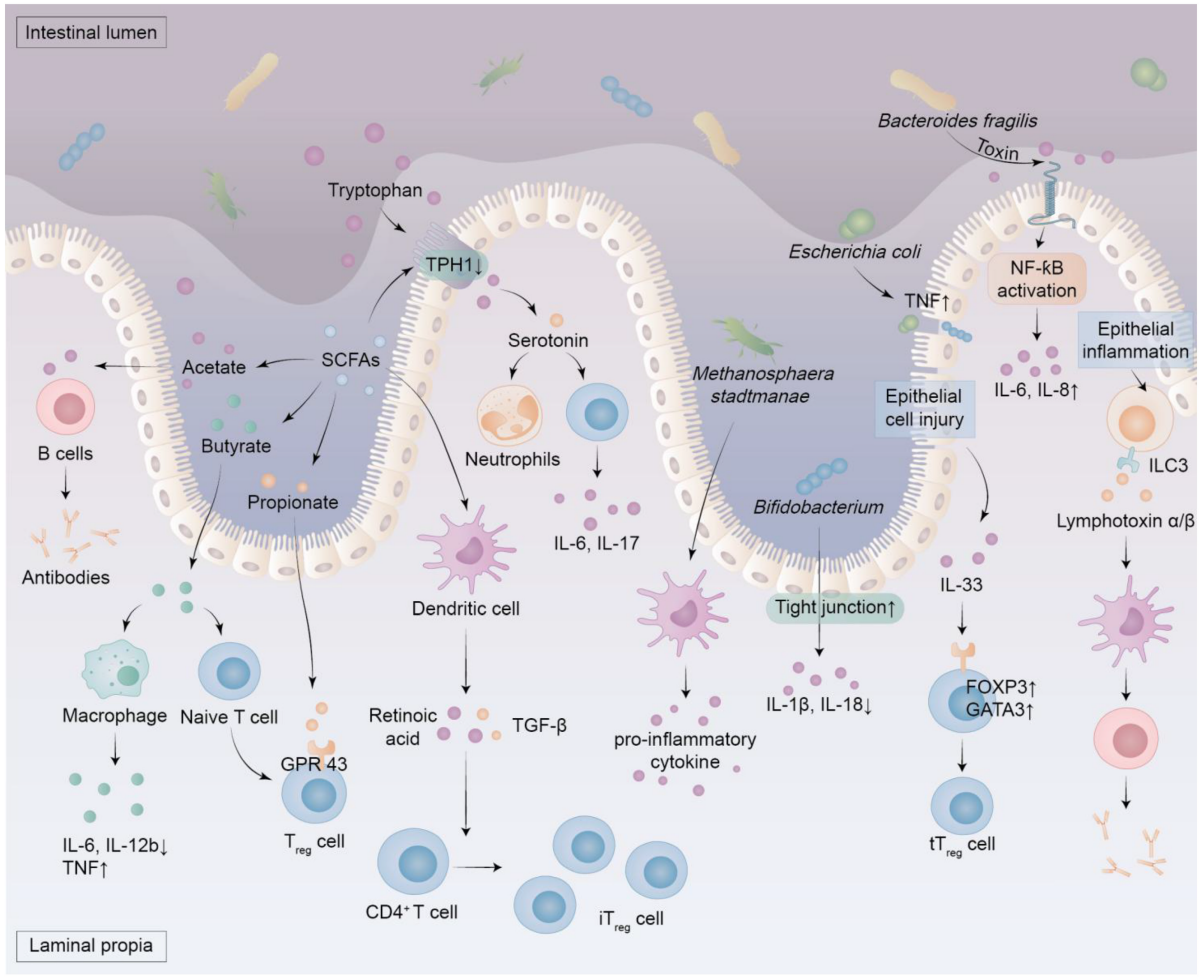
** The role of metabolites and components produced by the dysbiosis of intestinal microbiota.** Dysbiosis of the intestinal microbiota results in an elevation of pathogen levels and intestinal uremic toxins, which subsequently compromises the integrity of the intestinal barrier and instigates oxidative stress and inflammation within the intestinal mucosa. This process activates immune cells that secrete pro-inflammatory factors. Notably, certain components of this microbiota exacerbate the pro-inflammatory response by engaging the NF-ƙB signaling pathway. In cases of intestinal dysbiosis, there is a reduction in the production of SCFAs derived from dietary fiber fermentation by specific bacteria. This decline diminishes tryptophan hydroxylase 1 activity in enterochromaffin cells located within the gastrointestinal epithelium, resulting in decreased serotonin levels alongside increased tryptophan concentrations. Furthermore, SCFAs can activate dendritic cells to release TGF-β and retinoic acid, thereby facilitating the differentiation of CD4^+^ T cells into iT_reg_ cells. SCFAs include acetate, propionate, and butyrate, among others. Butyrate enhances macrophage polarization and anti-inflammatory responses by inhibiting TNF production while down-regulating IL-6 and IL-12b expression. It also promotes naive T cell differentiation into T_reg_ cells. Propionate stimulates GPR43 on T_reg_ cells, thereby augmenting their proliferation, whereas acetate fosters antibody synthesis in B cells. Inflammation of the epithelium induces ILC3 to produce lymphotoxin α and β, which leads to the activation of dendritic cells and helps B cells produce antibodies. Damage to epithelial cells prompts IL-33 release which increases GATA3 and FOXP3 expression in T cells, ultimately promoting both proliferation and maintenance of tT_reg_ cells. FOXP3, forkhead box protein 3; GATA3, GATA binding protein 3; ILC3, group 3 innate lymphoid cell; iT_reg_ cells, induced regulatory T cells; TPH 1, tryptophan hydroxylase 1; tT_reg_ cells, thymic regulatory T cells.

Most colonic bacteria are strict anaerobes that ferment indigestible complex carbohydrates into SCFAs, such as acetate, butyrate, and propionate (Figure 2) 49. Their proportions vary according to diet, with propionate generated by some species from Firmicutes and Bacteroidetes accounting for 10-20%, acetate generated by several bacterial groups accounting for 50-70%, and butyrate generated by a few *Clostridia* species constituting the majority of the remainder 50. Notably,* Mitsuokella jalaludinii* is an effective phytate degrader that can synergize with *Anaerostipes rhamnosivorans* to generate propionate 51. SCFAs play an important role in human health and disease 52. For instance, SCFAs produced by gut microbiota act as vital signalling molecules and metabolic substrates, impacting the host's metabolism, immune system, and gut-brain communication 52, 53. The SCFAs regulate extra-intestinal immunity directly and indirectly, and are associated with various disorders, including autoimmunity, infections, and intestinal inflammation 49, 52. The molecular mechanisms by which SCFAs regulate physiological processes involve the deorphanization of free fatty acid receptors 2 and 3 54. They regulate inflammation and barrier function via G protein-coupled receptors and histone deacetylase inhibition, with therapeutic potential in chronic inflammatory conditions 49. The SCFAs attenuate intestinal inflammation by striking a balance of regulatory T (Treg)/T helper 17 cells, while also lowering the levels of pro-inflammatory cytokines such as interleukin-6 (IL-6), IL-1β, and tumor necrosis factor-alpha (TNF-α) 55. The anti-inflammatory role of butyrate stems from direct modulation of phagocytes, intestinal epithelial cells, plasma cells, B cells, effector and Treg cells (Figure 2) 49. Due to its anti-inflammatory properties, high levels of butyrate are associated with improved intestinal immunity and intestinal barrier integrity, making it both nephroprotective and enteroprotective 56. In addition, propionate and butyrate serve as unique epigenetic regulatory elements linking diet, metabolism, and gene expression, and influence the proliferation of both normal cells and colorectal cancer cells 53. Overall, SCFAs serve as key mediators of the interaction between the host and the microbiota, and targeted supplementation with SCFAs may represent a promising therapeutic strategy for disease treatment 57.

#### 2.2.2. Microbial-derived tryptophan metabolites: AHR ligands

**Figure 3:**
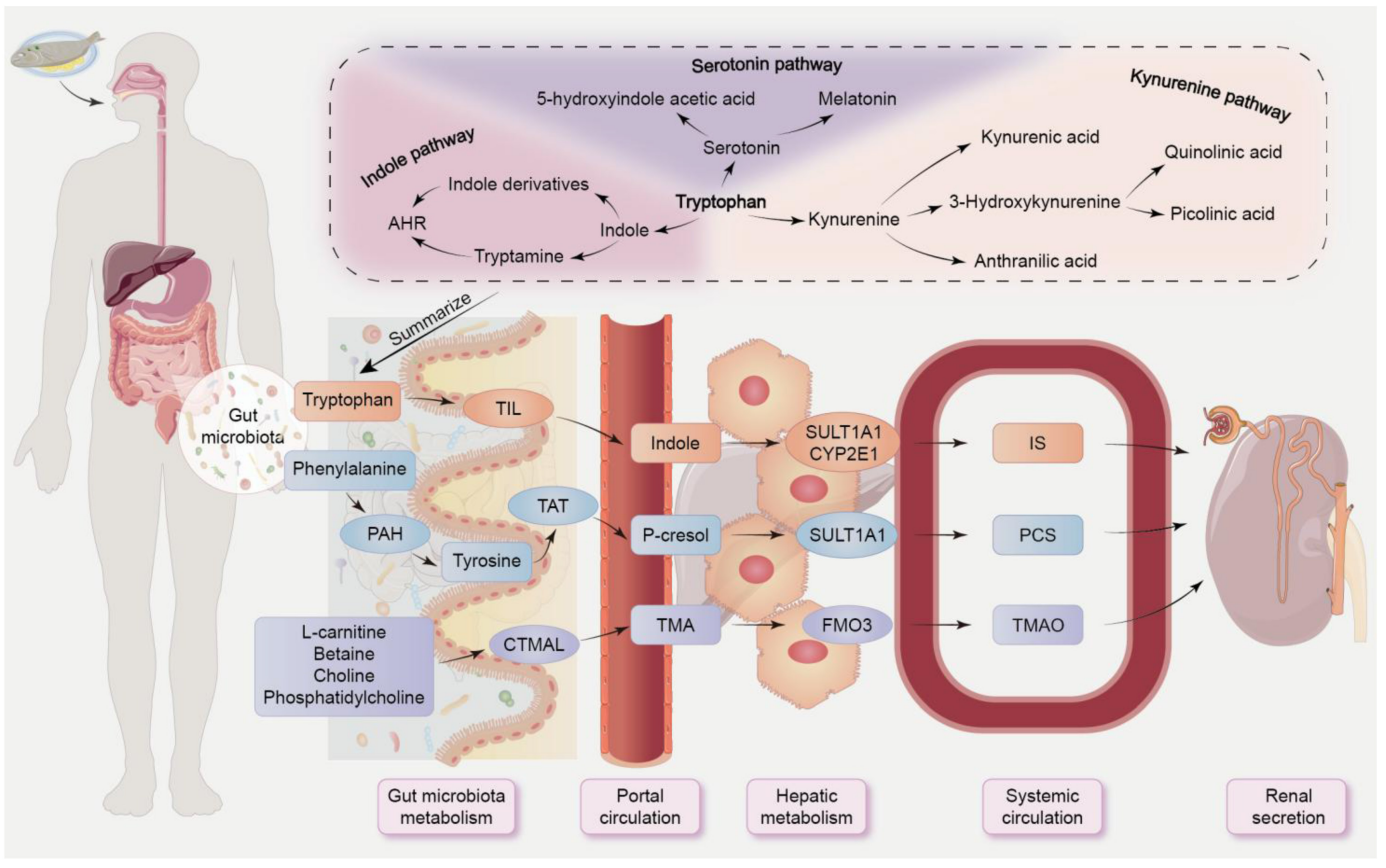
** The absorption and excretion pathways of gut-derived uremic toxins.** Some dietary breakdown products are excreted in faeces, while others enter portal circulation via efflux and influx transporters on colonocytes. The end products are then eliminated by healthy kidneys. Tryptophan can be catabolized by three different metabolic pathways: the serotonin pathway, the indole pathway, and the kynurenine pathway. In the indole pathway, tryptophan is converted to indole by TIL and subsequently absorbed into the portal circulation. Subsequently, it is metabolized to indoxyl sulfate by sulfotransferase 1A1 and cytochrome P450 2E1. Phenylalanine undergoes hydroxylation to form tyrosine through the action of intestinal bacteria utilizing PAH, with tyrosine further being metabolized to p-cresol via tyrosine aminotransferase. These intermediate metabolites are subsequently converted to p-cresyl sulfate by sulfotransferase 1A1. Choline and other dietary molecules are degraded into TMA through carnitine trimethylamine lyase, followed by oxidation to TMAO mediated by flavin-containing monooxygenase 3. CTMAL, carnitine trimethylamine lyase; CYP2E1, cytochrome P450 2E1; PAH, phenylalanine hydroxylase; TIL, tryptophan indole lyase; FMO3, flavin-containing monooxygenase 3; SULT1A1, sulfotransferase 1A1; TAT, tyrosine aminotransferase.

AHR, as a ligand-dependent transcription factor, regulates intricate transcriptional processes in diverse cell types and exerts primary anti-inflammatory and protective effects in the gut via innate lymphoid cells and IL-22 50, 58. Many tryptophan metabolites, such as IS, IAA, IPA and IAld produced by the gut microbiota, can act as AHR ligands, inducing AHR activation 58. Our previous research revealed that the plasma tryptophan level was negatively correlated with *Intestinimonas*, *Blautia*, and *Oscillibacter*, but positively correlated with *Lactobacillales*, *Pseudomonas*, *Turicibacter*, and *Clostridium IV* in rats with unilateral ureteral obstruction 48. Tryptophan can be catabolized through three different pathways: serotonin/melatonin pathway (≈1%-2%), indole pathway (≈5%), and kynurenine pathway (≈95%) (Figure 3) 32, 59. A small portion of dietary tryptophan is degraded in gut by bacterial tryptophan indole lyase to form indole, which is subsequently absorbed into portal circulation, and then converted to indoxyl, which is then transformed into indoxyl acetate and IS for excretion through the kidneys (Figure 3) 60, 61. Indole stimulates the release of GLP-1, while indole derivatives, including IAA, IAld, indole-3-acetaldehyde and indoleacrylic acid, can act as AHR agonists 50. Tryptophan supplementation reshapes gut microbiota, alleviates tissue damage, and enhances integrity of both intestinal epithelial and gut vascular barriers 62. Indole-3-lactic acid treatment protects the intestinal vascular barrier by downregulating claudin-2 via AHR/nuclear factor erythroid 2-related factor 2 (Nrf2)/signal transducer and activator of transcription 3-mediated pathways in mice induced by intestinal ischemia-reperfusion (I/R) 62. The latest study revealed that renal fibrosis is mediated by Wnt/β-catenin pathway via gut microbial-mediated tryptophan metabolism-driven AHR signaling 63.

#### 2.2.3. TMAO

TMAO is produced through hepatic processing of trimethylamine (TMA), which is derived from gut bacteria and is metabolised from certain dietary nutrients, such as choline, L-carnitine, betaine and phosphatidylcholine (Figure 3) 64, 65. *Emergencia timonensis* can catalyze the conversion of carnitine to the intermediate γ-butyrobetaine, which then converts to TMA, a precursor of TMAO 66. Plasma γ-butyrobetaine levels are significantly linked to incident cardiovascular disease (CVD) risk in a clinical cohort 66. Increasing evidence indicates that targeting uremic toxin-induced pathways or uremic toxins, including TMAO and tryptophan metabolites, may reduce the risk of CVD patients with CKD 67. TMAO, produced by liver and aorta, enhances trained immunity by increasing inflammation through endoplasmic reticulum stress, mitochondrial reactive oxygen species (ROS), and glycolysis pathways 68.

#### 2.2.4. PCS

PCS originates from the microbial metabolite p-cresol, which is derived from phenylalanine and tyrosine. Once absorbed into the portal circulation, p-cresol is converted by hepatic enzymes into harmful uremic toxins. The end-products of microbial protein fermentation are filtered out by tubular secretion and glomerular filtration (Figure 3) 69. Beyond renal excretion, the transport of PCS and IS, along with their microbiota-derived precursors p-cresol and indole, across key physiological barriers, including the blood-brain barrier, intestinal barrier, and renal proximal tubule, is also crucial for their elimination and distribution. Due to their high protein binding, PCS and IS are difficult to remove effectively by dialysis 70. The p-cresol has antioxidant and antimicrobial properties, but has also been linked to kidney disease, enteric pathogens, and autism 71. Molecular mechanisms of PCS include induction of apoptotic pathways, direct damage of cell membranes, activation of c-Jun N-terminal protein kinase and p38-mitogen-activated protein kinase, and activation of nicotinamide adenine dinucleotide phosphate oxidase 4 (NOX4) leading to ROS formation 72.

#### 2.2.5. Polyamines

The gut microbiota produces polyamines, including cadaverine, spermine, spermidine, and putrescine, which are essential for normal bacterial cell growth and multiplication 73. Polyamines exert anti-inflammatory effects by maintaining mitochondrial function and promoting autophagy, thereby conferring organ-protective and cytoprotective effects 74. Putrescine, which is derived from commensal bacteria, increases anti-inflammatory macrophages in the colon. As a substrate for symbiotic metabolism, putrescine is further absorbed and metabolized by the host, thereby contributing to the maintenance of intestinal mucosal homeostasis 75. Due to their polycationic nature, polyamines readily bind to anions in cells. Instead of binding to cytoplasmic proteins, intracellular polyamines primarily form polyamine-RNA complexes, stabilizing them 73. Small RNAs in extracellular vesicles derived from *Lactobacillus murinus* downregulate host polyamine metabolism by targeting enzymes involved in polyamine metabolism. Additionally, *Lactobacillus murinus* impeded the recovery of dextran sodium sulfate-induced colitis in mice by decreasing polyamine levels 76. Loss of spermine oxidase (SMOX), which produces spermidine, has been linked to increased α-defensin expression, exacerbated colitis, and microbiota dysbiosis by increasing abundance of Deferribacteres and Proteobacteria and reducing *Prevotella* abundance. Supplementing with spermine reverses these phenotypes, suggesting that it could be used as an additional treatment for colitis 77.

## 3. Microbial-derived metabolites as intermediates in gut-kidney communication

**Figure 4:**
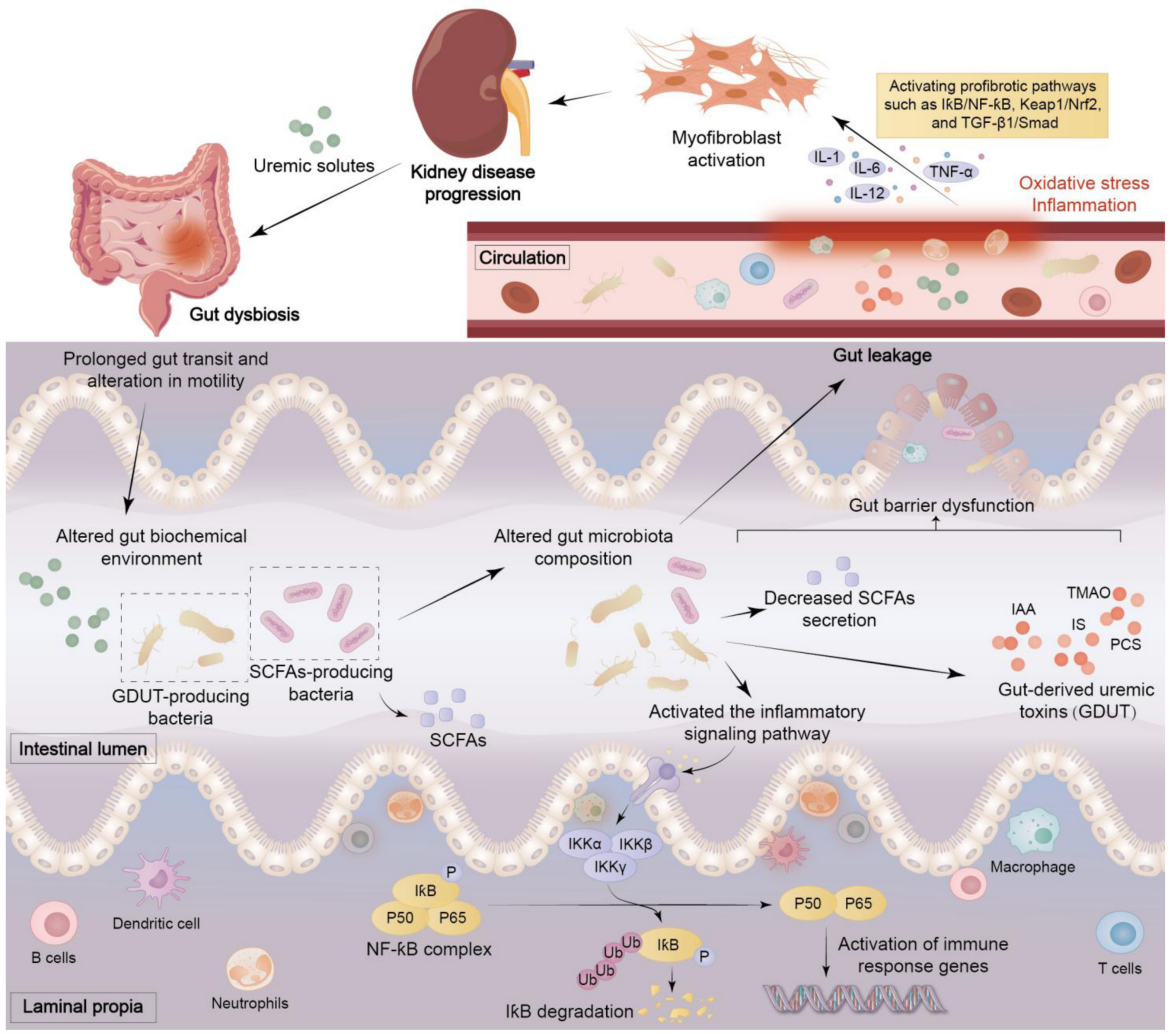
** The mechanisms of interaction between intestinal and kidney diseases.** Uremic solutes generated by kidney diseases can trigger gut bacteria dysbiosis, leading to prolonged intestinal transit time and altered peristalsis. The intestinal biochemical environment is then subject to alteration, affecting the composition of the intestinal microbiota, reducing the secretion of SCFAs, producing gut-derived uremic toxins (GDUT), and abnormally activating immune cells and inflammatory signalling pathways such as NF-ƙB. These factors have been demonstrated to induce intestinal barrier dysfunction, thereby precipitating leaky gut and bacterial translocation. Once uremic toxins enter the circulatory system, they trigger oxidative stress and inflammatory responses, activating profibrotic pathways such as IƙB/NF-ƙB, Keap1/Nrf2, and TGF-β1/Smad, prompting immune cells to secrete IL-1, IL-12, IL-6, and TNF-α, ultimately inducing renal fibrosis. This creates a vicious cycle that exacerbates kidney damage.

Renal disease can lead to dysbiosis of the intestinal microbiota, which can destroy the gut mucosal barrier and permit live bacteria or their metabolites to enter the bloodstream. The translocation and leakage of these metabolites across the intestinal barrier into the circulation trigger inflammation and oxidative stress, which ultimately lead to vascular and local tissue dysfunction 20, 32, 35. Renal disease leads to an increase in levels of uremic toxins originating from the colon. The features of the uremic microenvironment are intestinal microbial dysbiosis, intestinal epithelial cell barrier impairment, and immune cell dysfunction (Figure 4) 38. The uremic microenvironment leads to changes in the composition of microorganisms, infiltration of inflammatory cells, overproduction of immune complexes and antibodies, and abnormal activation of immune cells, which cause oxidative stress and inflammation that further exacerbate damage to renal parenchyma (Figures 2, 4) 48.

### 3.1. Intestinal epithelial cell barrier dysfunction in renal disease

AKI is a complex clinical syndrome that affects approximately 13.3 million people worldwide each year 78, 79. AKI is characterized by an abrupt drop in glomerular filtration rate (GFR), a reduction in urine output, a rise in serum electrolytes, urea and creatinine, which results in a rapid loss of kidney function and augments the risk of CKD 80, 81. AKI provokes intestinal inflammation and increased gut permeability 20. In pathophysiological conditions, proinflammatory cytokines, antigens and pathogens such as *Clostridium perfringens, Escherichia coli,* and *Vibrio cholerae*, cause intestinal epithelial cell barrier impairment 82. During AKI sepsis, the mucosal membrane barrier is disrupted, intensifying systemic inflammation and promoting aggravation of AKI 83. This suggests a potential link between intestines and kidneys (Figure 1). In severe acute pancreatitis, TNF-α elevates gut permeability, induces bacterial translocation from epithelium, ulteriorly irritates massive release of inflammatory cytokines, which probably exacerbate AKI 84.

CKD is a major global health problem that affects more than 10-15% of the adult population 85, 86. CKD is a progressive disease characterized by changes in the function and structure of the kidneys and is generally defined as persistent (>3 months) renal functional loss, high albuminuria or low GFR, eventually resulting in ESRD 87, 88. CKD patients show increased intestinal permeability and protective mucus layer degradation 89. Intestinal barrier dysfunction facilitates persistent leakage of metabolites through intestinal wall into systemic circulation and hepatic portal system, activating Toll-like receptors alongside inflammasome-mediated responses in innate immune cells as well as vascular endothelial cells across regional, local, and systemic sites 90. This triggers proinflammatory cytokine release and shedding of monocyte receptors, such as lipopolysaccharide (LPS) receptor, driving chronic inflammation typical of CKD 90. An analysis involving 48 children with CKD revealed stage-dependent increases in serum TNF-α, soluble CD14 and tryptophan metabolites alongside diminished production of SCFAs, suggesting gut barrier dysfunction, inflammation, and endotoxemia 91. Dodd *et al*. found that lower intestinal IPA impairs intestinal barrier integrity, elevating permeability and activating inflammation 92. As mentioned above, inflammatory factors and uremic toxins produced by gut microbiota during renal disease can lead to intestinal epithelial barrier dysfunction. Theoretically, gut microbial dysbiosis can compromise the health of this barrier.

### 3.2. Gut microbial dysbiosis in renal disease

#### 3.2.1 Gut microbial dysbiosis in AKI

Specific members within the intestinal microbial community may ameliorate or, conversely, exacerbate ischemic renal injury 93. I/R injury constitutes one of the primary causes of AKI 94. An increase in Enterobacteriaceae and a decrease in Ruminococcaceae and Lactobacilli were revealed to be hallmarks of I/R injury-induced dysbiosis and related to decreased levels of SCFAs, leaky gut, and intestinal inflammation 93. In LPS-induced AKI mouse models, pretreatment with the probiotic *Akkermansia muciniphila* substantially attenuated kidney injury and reduced tubular necrosis and apoptosis. This protective effect was accompanied by marked shifts in intestinal microbial ecology and preserved gut barrier integrity, demonstrating that modulating gut dysbiosis can ameliorate AKI outcomes 95. Therefore, the changes in the gut microbiota occur in the progression of AKI.

It is worth noting that AKI progression also affects the composition of the gut microbiota. Clinically, AKI-induced gut microbiota dysbiosis includes disturbances in *Bifidobacterium*,* Bacteroidetes*, *Salmonella*, *Staphylococcus*, and* Faecalibacterium* 93. Another study indicated that the abundance of* Bifidobacterium* was decreased while the abundance of* Ruminococcus* and *Clostridium* was increased after I/R injury 96. In renal I/R injury models, AKI was found to downregulate enzymes in the intestinal transsulfuration pathway, reduce glutathione levels, impair tight junctions, and disrupt gut microbiota composition 97. In summary, kidney damage can alter the structure and composition of the gut microbiota.

#### 3.2.2 Gut microbial dysbiosis in CKD

The gut microbiota contributes to the progression of CKD by producing uremic toxins and inducing systemic inflammation 98. The accumulation of bacterial species that produce uremic toxins over time was associated with worsening renal function. The abundance of these bacterial species was significantly higher in patients with severe CKD than in those with moderate CKD 99. Additionally, *Bacteroides fragilis* has been shown to mitigate renal fibrosis by increasing 1,5-anhydroglucitol concentrations and decreasing LPS levels 34. A prominent characteristic of diabetic kidney disease (DKD) is a lower ratio of Firmicutes (gram-positive) to Bacteroidetes (gram-negative), which positively correlates with plasma glucose levels 100. Notably, the relative abundance of *g_Prevotella_9* can accurately predict individuals with diabetes 69. In diabetic mice, the presence of gut-derived *Klebsiella oxytoca* in the kidneys and circulation, alongside elevated IL-17, was found to induce the expression of kidney injury molecule-1, thereby promoting DKD progression 101. The significant expansion of the *Escherichia-Shigella* genus may serve as therapeutic targets and promising diagnostic biomarkers for immunoglobulin A nephropathy (IgAN) 102. Notably, the abundance of *Bifidobacterium* was decreased in both IgAN patients and mice, and the proportion of *Bifidobacterium* was negatively correlated with hematuria and proteinuria levels, suggesting that reduced *Bifidobacterium* abundance might be associated with IgAN severity 103. A negative correlation was revealed between the abundance of* Escherichia-Shigella* and proteinuria levels, while the abundance of *Bacteroides* and *Klebsiella* was positively correlated with membranous nephropathy (MN) stage 39. Moreover, the increase of pathogenic bacteria and the reduction of *Roseburia* and certain species of *Faecalibacterium* that can ferment various sugars into SCFAs may cause or exacerbate primary MN 104.

The uremic environment caused by CKD, characterized by elevated blood urea levels, metabolic acidosis, and systemic inflammation, can alter the composition of the gut microbiota. CKD patients exhibited reduced microbiota diversity, altered community structures, disrupted potential functions and microbial composition 30, 105. Mollicutes and Tenericutes were enriched in CKD patients at tages 1-2, *Parasutterella* was enriched in CKD patients at stages 3-4, and *Blautia* and *Akkermansia* were enriched in CKD patients at stage 5 106. Ren *et al*. found that the abundances of *Akkermansia* and *Thalassospira* were increased with CKD progression 106. Wu *et al*. discovered that a core CKD-related microbiota consists of 7 bacteria genera and 2 bacteria species including *Bacteroides eggerthii* and *Collinsella stercoris*, which are associated with the stages of CKD 107. The discriminative species for DKD patients with microalbuminuria include *Acinetobacter johnsonii* and *Escherichia coli*, whereas for DKD patients with macroalbuminuria, *Enterococcus faecalis*, *Morganella morganii*, and *Pseudomonas_E oleovorans* were identified 108. Studies have shown that the dysbiosis observed in patients with CKD is not static, but worsens progressively as the disease advances. Furthermore, over time, the proportion of uremotoxin-producing bacteria gradually increased, while the overall abundance of microbiota decreased 99. This suggests that declining renal function directly drives the dynamic evolution of the gut microbiota composition. In summary, renal disease and gut microbial dysbiosis create a vicious cycle that exacerbates each other and accelerates disease progression.

### 3.3. Microbial-derived metabolites in renal disease

#### 3.3.1. SCFAs

Recent studies have revealed that intestinal microbiota-associated metabolites, particularly SCFAs, are closely correlated with renal disease 109. *L. casei Zhang* increased nicotinamide and SCFAs levels in the serum and kidneys of mice with bilateral renal I/R, mitigating renal damage and slowing the progression to CKD 41. Treatment with three major SCFAs (acetate, propionate, and butyrate) improved renal dysfunction in mice with I/R, associated with reduced levels of local and systemic inflammation, cell activation/infiltration, and apoptosis, increased autophagy, and oxidative cellular stress 110. Treatment of mice with acetate-producing bacteria also resulted in improved outcomes following AKI 110.

**Figure 5:**
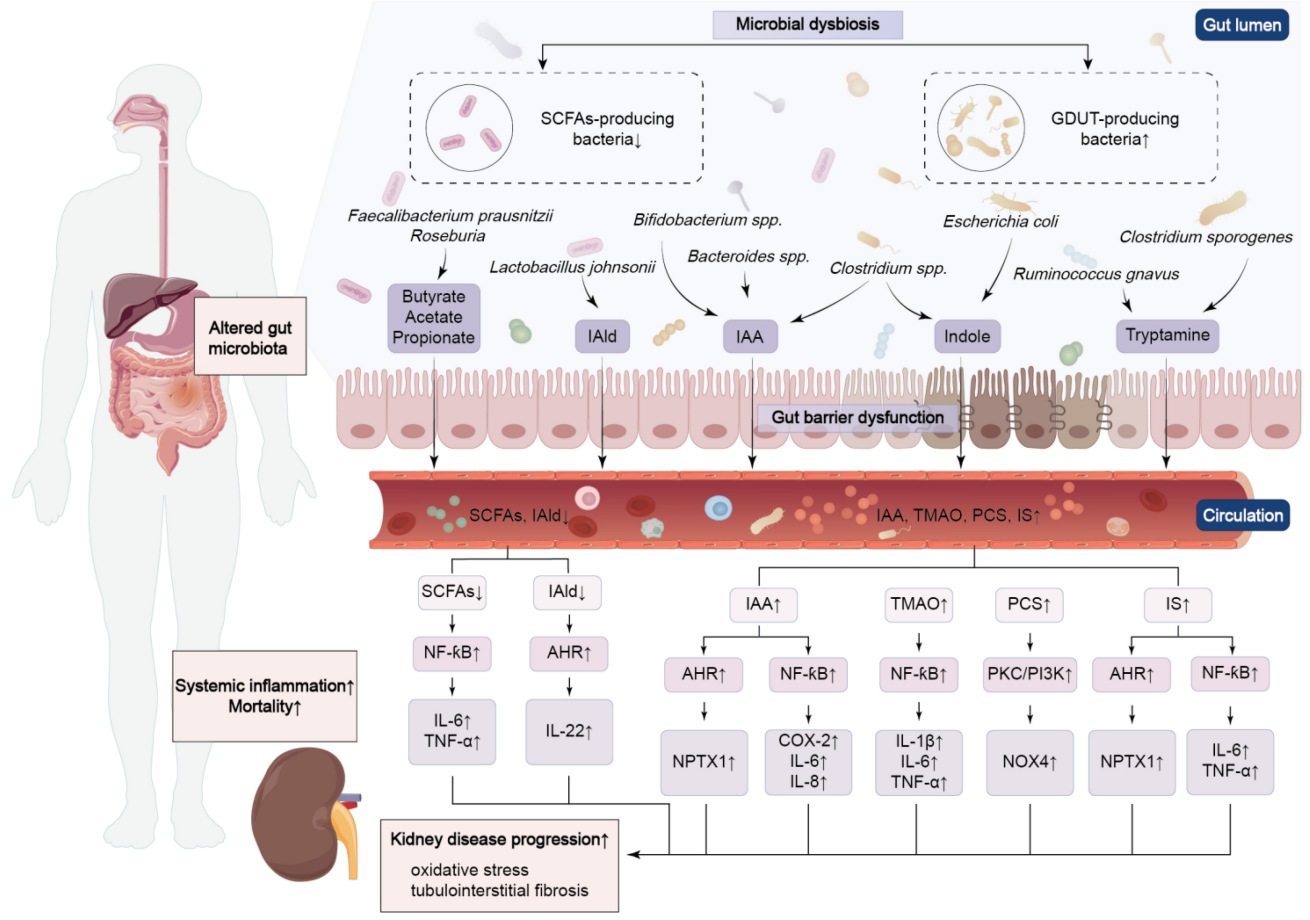
** The molecular mechanism of microbial dysbiosis-mediated renal disease.** During renal disease, pathogenic bacteria increase and beneficial bacteria decrease, causing metabolite disorders. These metabolites can activate the AHR receptor or NF-ƙB signalling pathway directly or indirectly, thereby exacerbating kidney disease. NPTX1, neuronal pentraxin 1; PKC, protein kinase C.

It was found that *Faecalibacterium prausnitzii*, a bacterium that produces butyrate and has anti-inflammatory properties, was depleted in CKD patients during its progression to ESRD. Consequently, butyrate levels decreased and intestinal homeostasis was disrupted 111. Butyrate partially ameliorates renal function via G-protein-coupled receptor 43 (GPR43)-mediated suppressing oxidative stress and NF-ƙB signaling pathway (Figure 5) 31, 111. Administering *L. casei Zhang* orally can slow the decline in kidney function in patients with stage 3-5 CKD by modifying nicotinamide and SCFAs metabolism, thereby alleviating kidney damage and delaying the progression of renal decline 41. *Gemmiger formicilis* is a chemoorganotrophic, gram-negative bacterium that synthesises butyrate via the acetyl coenzyme A pathway. The butyrate derivative sodium butyrate shows potential as a therapy for DKD, which suggests that *Gemmiger* may have nephroprotective properties 112. Similar to humans, the diabetic rodent model exhibited dysbiosis, characterised by decreased α-diversity and increased abundance of Actinobacteria and Firmicutes, as well as a loss of SCFAs-producing bacteria such as *Bacteroides*, *Rikenella*, and *Ruminococcus* 113. Perinatal propionate supplementation reverses CKD-induced hypertension in offspring of female rats. This protective effect correlates with increased plasma propionate and propionate-generating bacteria *Clostridium* spp. levels, enhanced expression of the renal GPR41 114.

Reduced SCFAs are not merely correlates but causal drivers of renal fibrosis. In a preclinical mouse model of the transition from AKI to CKD, administration of butyrate and propionate both prevented disease progression and downregulated pro-fibrotic genes, demonstrating therapeutic potential independent of the intestinal microbiota 115. In adenine-induced CKD mouse models, supplementation with butyrate effectively alleviated renal fibrosis, potentially by modulating nucleotide-binding oligomerization and domain-like receptor family protein 3 (NLRP3)-mediated pyroptosis through the Stimulator of interferon genes/NF-ƙB/p65 pathway 116. Butyrate supplementation decreases the overexpression of TGF-β, which is induced by persistent, low-grade inflammation. It also suppresses the dilatation of glomerular and mesangial areas, oxidative damage and interstitial fibrosis in DKD mice through Nrf2-dependent mechanisms 31. It is worth noting that the effects of SCFAs in inhibiting renal fibrosis and mesangial matrix accumulation are primarily mediated by GPR43 overexpression or GPR43 agonists. This confirms that SCFAs, particularly butyrate, may improve renal fibrosis through GPR43-mediated suppression of oxidative stress and NF-κB signaling 117. Therefore, supplementing SCFAs may represent a promising direction for future research on renal fibrosis.

#### 3.3.2. Tryptophan metabolites: AHR ligands

The accumulation of tryptophan metabolites in patients with renal failure is due to alterations in enzyme activity, gut microbial dysbiosis, and inefficient renal excretion, which ultimately resulting in an increase in tryptophan catabolites in intestine 67. A study involving 63 patients with sepsis found that amino acid metabolism, which adjusts immunity and inflammation, was commonly dysregulated 118. The biosynthesis of tryptophan and tyrosine was associated with the key molecular alterations within the multiomics network in sepsis. Phenylalanine metabolism was found to be associated with sepsis-related AKI 118. It has been demonstrated that AHR activation in kidney disease is both tissue- and time-dependent. Using transgenic mouse models, Walker *et al*. showed that adenine-induced CKD not only leads to significant AHR activation in renal tubules, but also in hepatocytes, cardiac myocytes, and cardiac myocytes, hepatocytes, and microvasculature in the cerebral cortex. They also demonstrated that this activation pattern is closely correlated with serum IS levels 119. In I/R-induced AKI, AHR activation in the renal tubules persists even after serum uremic toxin levels have decreased, suggesting the presence of other local injury-related mechanisms 119. Intestinal bacterial translocation and low levels of endotoxemia, alongside decreased levels of proline, tryptophan, and tyrosine, have been observed in mice with renal I/R injury 96. *Bifidobacterium longum* modulated macrophage inflammasome formation and mitochondrial fatty acid oxidation via histone deacetylase 3-mediated indole-3-carboxaldehyde, thereby counteracting intestinal I/R in mice 120. Furthermore, Balestrieri *et al*. revealed that AHR activation in muscle exacerbates ischaemic pathology in CKD and that AHR activation in muscle is a key regulatory factor in ischaemic limb pathology in CKD 121. Muscle-specific AHR knockout improved limb perfusion and preserved muscle mass and mitochondrial function, whereas constitutive AHR activation resulted in myopathy 121. Therefore, gut-derived tryptophan metabolites that act as AHR ligands not only drive renal fibrosis, but also extra-renal complications of CKD.

A study of patients with stage 1-5 CKD found *Lactobacillus johnsonii* (*L. johnsonii*) supplementation attenuated renal lesions by restraining AHR signaling via augmented serum IAld levels 36. A recent study revealed that the combination of AHR and IAld enhances the production of IL-22 by lamina propria lymphocytes, triggering the phosphorylation of transduction factor 3 and signal transduction factor. This stimulated the proliferation of mouse intestinal epithelial cells and repairs intestinal mucosal injury (Figure 5) 122. Previous several studies indicated that IAld acted as a ligand of AHR and reduced levels of IL-1β, TNF-α, monocyte chemoattractant protein-1 (MCP-1), and cytochrome P450 family 1 subfamily A member 1 in mice 123, 124. In adenine-induced CKD rats, serum IAld levels demonstrated a strong negative relationship with creatinine levels, confirming that *L. johnsonii* supplementation ameliorates renal lesions 36.

Gut microbiota that produces uremic toxin precursors were found to be more prevalent in CKD patients than in healthy controls 99. Therefore, uremic toxins, including IS and IAA, accumulated progressively in the course of CKD 30, 125. IS-mediated AHR binding to NF-ƙB p65 subunit, resulting in mutual inhibition of NF-ƙB and AHR, which inhibited the secretion of IL-6 and TNF-α in macrophages of CKD patients 126. Serum IAA has been identified as a potential independent predictor of CVD and mortality in CKD patients, due to its ability to induce endothelial inflammation and oxidative stress, increase cyclooxygenase-2, IL-6, and IL-8 levels, and activate the inflammatory AHR/p38-mitogen-activated protein kinase/NF-ƙB pathway (Figure 5) 127. Two species, *Fusobacterium nucleatum* and *Eggerthella lenta*, increased uremic toxins production in the adenine-induced CKD rats, with the former producing phenol and indole and the latter producing hippuric acid, which promote kidney damage 40. Moreover, IAA and IS upregulate expression of neuronal pentraxin 1, which belongs to the inflammatory protein family, in an AHR-dependent manner (Figure 5) 128.

Recent studies have established causal links between tryptophan metabolites and renal fibrosis. Xie *et al*. showed that IS activated AHR to promote renal senescence and fibrosis via receptor gamma coactivator 1-α degradation, and AHR knockout abolished this effect 129.Intrarenal 1-methoxypyrene, an endogenous AHR agonist, accumulated progressively in obstructed kidneys and directly mediated tubulointerstitial fibrosis via the AHR. AHR knockdown or the use of specific AHR antagonists could attenuate 1-methoxypyrene-induced fibrosis 17. Convergently, targeting *Lactobacillus johnsonii* in CKD patients and animal models increased serum IAld, a protective tryptophan metabolite that suppressed AHR signaling and reversed established kidney lesions 36. A reduced relative abundance of *Bifidobacterium animalis*, *Lactobacillus murinus*, *Lactobacillus johnsonii*, *Lactobacillus reuteri*, and *Lactobacillus vaginalis* was found to be positively correlated with decreased tryptamine, indole-3-pyruvic acid, and IAld levels, and negatively correlated with increased IAA and indole-3-lactic acid levels in the serum of MN rats 39. In summary, these findings suggest a causal relationship between tryptophan metabolites produced by the gut microbiota and the progression of renal fibrosis.

#### 3.3.3. TMAO

TMAO was identified as a key metabolite linked to the AKI-to-CKD transition in both AKI patients and AKI-to-CKD transition mice 130, 131. *In vivo* and *in vitro* studies confirmed that NOX2 activation was a critical regulator of TMAO-related transition from AKI to CKD 131. TMAO has been shown to cause kidney damage and tubulointerstitial fibrosis, and elevated plasma concentrations of TMAO in CKD patients predict poor long-term survival 132, 133. Among community-dwelling US adults, higher serial plasma TMAO levels were linked to a higher risk of incident CKD and a greater annual decline in kidney function 132. The advent and progression of TMAO-mediated CKD principally comprise inflammatory mechanisms. A clinical study confirmed that DKD patients with higher plasma TMAO levels had poorer renal outcomes and a higher mortality rate. TMAO activated the NF-ƙB pathway in DKD patients, exacerbating systemic microinflammation and accelerating DKD progression 100. TMAO increased renal scarring via TGF-β1 signaling pathways, and circulating TMAO levels were substantially reduced in patients receiving hypoglycemic drugs with reno-protective properties, such as GLP-1 receptor agonists 134. Nondialysis patients with stage 3-5 CKD who exhibited rapid estimated glomerular filtration rate (eGFR) decline possessed a unique intestinal microbial composition, characterized by elevated α-diversity and an abundance of TMA-producing bacteria, such as *Collinsella tanakaei* and *Desulfovibrio* 135. *In vivo* studies suggest that TMAO activates the NOD-like receptor protein 3 inflammasome, leading to the secretion of IL-18 and IL-1, and exacerbating renal inflammation and fibrosis (Figure 5) 31. Targeting macrophage phenotype through gut-kidney axis improved renal fibrosis in mice. In both *in vitro* and *in vivo* culture experiments, TMAO stimulation was found to increased mRNA expression for IL-1β, IL-6, nitric oxide synthase, and TNF-α 136. TMAO worsens kidney damage caused by hyperoxaluria in mice by activating the protein kinase R-like endoplasmic reticulum kinase/ROS pathway. This boosts apoptosis, autophagy, and inflammation, while also promoting calcium oxalate crystals accumulation in renal tubular cells 137. These findings suggest that TMAO shows great potential as a therapeutic target for treating renal fibrosis, and we plan to develop strategies for treating renal disease by modulating TMAO levels.

#### 3.3.4. PCS

*Lactobacillus salivarius* BP121 prevented cisplatin-induced AKI in rats by modulating the intestinal environment and reducing inflammation and oxidative stress, thereby inhibiting PCS and IS production, while regulating AMP-activated protein kinase- and Toll-like receptor 4 (TLR4)-dependent tight junction protein assembly 138. With the advancement of CKD, phenylalanine, tyrosine, and tryptophan metabolism is enhanced within uremic toxin-related metabolic pathways, in contrast, proline and arginine metabolism is weakened 106. PCS is a protein-binding uremic toxin that accelerates the deterioration of renal function in CKD patients and raises the risk of CVD. Microbiota-driven therapies, such as synbiotics or prebiotics, can reduce circulating PCS concentrations in patients with CKD 139. A study of urine and serum samples taken from 90 DKD patients in different stages revealed that urinary PCS and serum metabolites such as butenoylcarnitine, arginine, and IS may serve as biomarkers of early DKD 140. Freise *et al*. found that the effects of gut-derived uremic toxins (PCS, TMAO, and IS) on glycosaminoglycans involve the activation of the transcription factor NF-ƙB and of the phosphatidylinositol-3 kinase (PI3K)/protein kinase B pathway 141. PCS activates ROS production and nicotinamide adenine dinucleotide phosphate oxidase through the protein kinase C and PI3K signalling pathways, triggering the expression of inflammatory cytokines in 5/6-nephrectomized rats (Figure 5) 142. Moreover, Wu *et al*. also found that reduced* Prevotella sp. 885* correlated with urea excretion, while elevated PCS inversely correlated with GFR 143.

PCS can directly induce renal tubular damage and fibrosis. Administration of PCS for four weeks in 5/6-nephrectomized rats caused significant tubular injury and oxidative stress 142. Knockdown of NOX4 suppressed PCS-induced pro-oxidant effects, and probenecid blocked its toxicity, confirming that intracellular accumulation and NADPH oxidase activation are required for its profibrotic action 142. Taken together, modulating gut-derived uremic toxins PCS may provide an additional effective therapeutic strategy for treating kidney disease.

#### 3.3.5. Polyamines

Sepsis-induced AKI in mice was prevented by spermidine, which decreased NLRP3 inflammasome activation and IL-1β production 144. In CKD rats, increased acrolein, spermidine, spermine, N1,N12-diacetylspermine, and N1-acetylspermidine were associated with an increase in *Escherichia_Shigella*, *Clostridium_sensu_stricto*, *Parasutterella*, and *Enterorhabdus*, while increased putrescine was linked to *Bacteroides* 145. Mice with CKD following 5/6 nephrectomy exhibited reduced fecal output, with this constipation associated with suppressed ileal contractile responses. Spermine was found to suppress ileal contractile responses 146. Taken together, microbiome-derived metabolites are altered in kidney disease and can influence the progression of the disease.

The development of renal fibrosis is influenced by polyamine metabolism. Causal evidence establishes polyamine metabolism as a determinant of renal fibrosis. Zahedi *et al*. demonstrated that ablation of polyamine catabolic enzymes significantly reduced cisplatin-induced renal injury and fibrosis, proving that enhanced polyamine catabolism drives fibrotic progression 147. In contrast, Nakano *et al*. showed that spermidine activates Nrf2 and suppresses TGF-β1 signaling and oxidative stress, and spermidine treatment prevents fibrotic progression in arginase 2 knockout mice, establishing a protective causal pathway 148. The expression of SMOX, a vital enzyme that regulates polyamine metabolism, is positively associated with renal fibrosis and function decline in CKD patients 149. Luo *et al*. discovered that the SMOX/spermine axis could be a new, promising therapeutic approach for counteracting renal fibrosis, potentially by suppressing senescence and coordinating autophagy 149. Therefore, polyamines influence the progression of renal fibrosis, and targeting gut microbiota may be an effective strategy for patients to slow the progression of renal disease. Many therapeutic strategies currently target this critical aspect.

#### 3.3.5. Differential gut microbial and metabolite in renal disease

Studies have demonstrated significant differences in the dysbiosis of gut microbiota and metabolites between AKI and CKD, as well as between early and advanced stages of CKD. Plasma TMAO has been identified as significantly associated with an increased risk of AKI-to-CKD transition. NOX2 activation has been confirmed as a key regulator mediating TMAO-related AKI-to-CKD progression, both *in vitro* and *in vivo* 131. Furthermore, plasma TMAO levels in CKD patients can predict long-term mortality and cardiovascular events, highlighting its prognostic value 150. A large population-based cohort study demonstrated that PCS, IS, and phenylacetylglutamine were independently and inversely associated with eGFR, serving as early markers of renal function decline 151. Propionate was substantially lower in advanced CKD and exhibited high discriminative power between patients with advanced CKD and non-CKD controls 143. These investigations indicate that specific microbial by-products might act as early alerts for the advancement of AKI to CKD or as indicators for the severity of CKD.

In CKD, progressive alterations in gut microbial composition and metabolite profiles correlate with disease severity. Wu *et al*. found that the levels of six circulating metabolites and thirteen microbial species changed significantly from early to advanced CKD. Among them, *Bacteroides eggerthii* and* Prevotella sp. 885* were the most effective features for classifying mild and advanced CKD, respectively, and *Bacteroides eggerthii* was particularly valuable for early diagnosis 143. A core CKD-associated microbiota comprising seven genera and two species (*Bacteroides eggerthii* and* Collinsella stercoris*) was discovered to be highly correlated with CKD staging. Notably, *Collinsella stercoris*,* Paraprevotella*, and* Pseudobutyrivibrio* could distinguish early CKD from controls with better discriminatory performance than the conventional urine protein-to-creatinine ratio 107. In the PREDIMED-Plus trial, lower abundances of the butyrate-producing genera *Lachnospira* and* Lachnoclostridium* were associated with CKD progression 152. Recent studies revealed that patients with severe CKD harbored a higher enrichment of uremic toxin-producing species compared with those with moderate CKD. Furthermore, toxin-producing bacterial species gradually increased over time, whereas microbial diversity progressively decreased 153.

Collectively, these findings indicate that gut microbial dysbiosis evolves dynamically with the progression of CKD, characterized by the enrichment of uremic toxin-producing bacteria and the depletion of SCFAs-producing bacteria, accompanied by differential changes in metabolites such as TMAO. Thus, these factors hold potential as biomarkers for monitoring disease progression.

## 4. The therapeutic strategies in renal disease by modulating gut microbial-derived metabolites

The understanding of underlying molecular mechanisms in disease aims to discover novel therapeutic agents and strategies. The mentioned-above studies indicate that the changes in gut microbial-derived metabolites in kidney disease including both AKI and CKD. Based on the elucidation of underlying molecular mechanisms, increasing studies suggest that a number of therapeutic strategies, such as dialysis (hemodialysis, peritoneal dialysis and colonic dialysis), KT, natural product interventions, microecological therapies (probiotics, prebiotics and synbiotics), and dietary interventions, enhanced kidney function and improved renal injury by regulating gut microbial-derived metabolites 154, 155. Therefore, targeting gut microbiota has been demonstrated as an effective and promising therapy for treating kidney diseases.

### 4.1 Regulating gut microbial-derived metabolites by renal replacement therapies

In the last several decades, extensive clinical data have shown renal replacement therapies including KT and dialysis, such as hemodialysis, peritoneal dialysis and colonic dialysis, is the final treatment for patients with ESRD and uremia that improve life quality 156-158. Increasing publications have revealed that these therapies can affect the levels of the gut microbial-derived metabolites, such as IS, PCS and TMAO and regulated pro-inflammatory cytokines and AHR signalling pathway 91, 159.

#### 4.1.1 Regulating microbial-derived metabolites by hemodialysis and peritoneal dialysis

Hemodialysis (HD) is a commonly used dialysis method for patients with ESRD and uremia worldwide 160-162, while peritoneal dialysis (PD) is also widely used for the long-term treatment of ESRD, accounting for 9% of all renal replacement therapy and 11% of all dialysis globally 154, 163. PD is a renal replacement therapy for ESRD, and is now commonly used to achieve target clearances for creatinine or urea 164. Lin *et al*. demonstrated that distinct gut microbial composition was linked to an elevated mortality risk in HD patients, and that the abundance of *Anaerostipes* and *Succinivibrio*, two SCFAs-producing bacteria, was markedly lower in non-survivors 155. Changes in the microbiome of CKD patients are phase-dependent, being most pronounced in HD, with a decrease in saccharolytic bacteria, such as *Bifidobacterium*, and an increase in proteolytic bacteria, such as *Citrobacter* 91. HD patients showed lower plasma tryptophan levels but higher IS and five kynurenine metabolites, including anthranilic acid, kynurenic acid, kynurenine, xanthurenic acid, and 3-hydroxykynurenine 91. Since kynurenine and IS can activate AHR, serum-induced AHR activity was higher in HD patients than healthy controls 91. HD patients have higher levels of serum IS and PCS than healthy controls. These protein-bound uremic toxins are difficult to remove by dialysis and are key factors in the development of CVD 165. TMAO, a gut microbial metabolite, raises the risk of peritoneal inflammation and peritonitis in PD patients. *In vitro* study demonstrated that TMAO directly triggered necrosis of primary peritoneal mesothelial cells, alongside elevated production of pro-inflammatory cytokines, including IL-6, IL-1β, TNF-α, and MCP-1. Furthermore, TNF-α-induced P-selectin generation in mesothelial cells was considerably increased by TMAO 159. Both HD and PD patients exhibited increased levels of proinflammatory cytokines, such as TNF-α and IL-6 155, 166. IL-6 has been revealed to affect the structural and inflammatory properties of the peritoneal membrane 166. HD patients exhibit myeloid cells alters, shown by a shift from classical to non-classic and proinflammatory intermediate monocytes, which efficiently produce TNF-α 91. Collectively, these findings suggest that both hemodialysis and peritoneal dialysis affect the gut microbial-derived metabolites in CKD patients.

#### 4.1.2 Regulating microbial-derived metabolites by colonic dialysis

Colonic dialysis has long been employed in China to remove intestinal-derived uremic toxins and delay CKD progression. The injection of an osmotically balanced solution into the colon during colonic dialysis flushes out persistent stool and intestinal-derived uremic toxins 167. Colonic dialysis ameliorated renal function in patients with stage 3-5 CKD and increasesd the abundance of anaerobic bacteria producing SCFAs in CKD patients, including *Bifidobacterium*, *Bacteroides*, and *Collinsella*, while reducing the abundance of microbes that possess urease and generate PCS or IS, including unclassified Enterobacteriaceae 167, 168. A 20-year-old female patient with ESRD underwent colonic dialysis via a Malone antegrade continent enema stoma for two years. Throughout this period, her serum blood urea nitrogen (BUN) and creatinine levels remained consistently low, suggesting that this approach could be a promising alternative to renal replacement therapy 169. Considerable decreases in creatinine and BUN levels were observed following colonic dialysis in uremic rats that had undergone left partial nephrectomy and right nephrectomy 170. Clinical evidence suggests that a combination of colonic dialysis and traditional Chinese medicine enemas can treat chronic renal failure by reducing Scr levels 171. Colonic dialysis using Chinese medicine altered creatinine decomposition by gut bacteria in uremic rats, reducing *Lactobacillus* and *Bifidobacterium* abundance and increasing *E.coli* abundance in the colon 172. Colonic dialysis with Gubenxiezhuo significantly improved apoptosis and inflammatory infiltration in uremic rats, reduced hemorrhage, and attenuated the expression levels of C-reactive protein, IL-6, and IL-1β 173. Therefore, colonic dialysis may be a promising approach to treating kidney disease in the future.

#### 4.1.3 Regulating microbial-derived metabolites by KT

Compared to dialysis, KT offers a higher survival rate and improves life quality, making it an advantageous treatment option for patients with ESRD 174-176. Compared to PD or HD, KT reduced levels of several uremic toxins and also reduced the risk of CVD 67, 177. Swarte *et al*. found that a lower abundance of four butyrate-producing bacteria, including *Eubacterium hallii*, *Firmicutes bacterium CAG 83*, *Faecalibacterium prausnitzii*, and *Gemmiger formicilis*, was linked to a higher risk of death. KT patients had lower butyrate-producing bacteria abundance than healthy controls. Lower levels of these bacteria also linked to poorer health-related quality of life in KT patients 178. Metabolites like kynurenine, polyunsaturated fatty acid, uric acid, creatinine were identified as differential serum metabolites in acute post-transplant graft rejection 179. Although TMAO levels in KT recipients declined sharply, they did not reach the levels seen in healthy controls. Furthermore, an increased mortality risk was independently associated with elevated plasma TMAO concentrations in KT patients 180. A high-fiber diet or sodium acetate supplementation altered the microbiome, modifying alloimmunity in a mouse KT model. This generated tolerance dependent on GPR43 and Tregs 181. Collectively, these findings emphasize complex interplay between metabolic dysregulation, gut microbiota, and overall health in patients receiving renal replacement therapy.

### 4.2 Regulating gut microbial-derived metabolites in renal disease by natural products

Traditional Chinese medicines have been long practiced and are commonly recognised as an essential treatment for various kidney diseases including AKI and CKD 182-189. Recent studies emphasize natural products improved AKI and CKD by regulating the dysbiosis of gut microbiota and the disorder of gut microbial-derived metabolites 36, 39, 190.

#### 4.2.1 Regulating gut microbial-derived metabolites in AKI by natural products

A larger body of research study has demonstrated that natural products exhibit a significant therapeutic effect in ameliorating AKI 191-195. Studies have demonstrated that natural compounds exhibit obvious therapeutic effects on animal models with renal disease. Total alkaloids of *Aconitum carmichaelii* Debx (ACA) retarded cisplatin-induced AKI by decreasing relative abundance of *Escherichia-Shigella*, *Ruminococcus*, and *Clostridium*, while increasing *Bacteroides*,* Ligilactobacillus*,* Desulfovibrio*, and *Anaerotruncus* 196. This were accompanied by reducing uremic toxins, and elevating serum levels of SCFAs that were related to the glutathione and tryptophan metabolism, decreasing serum levels of inflammatory factors, such as IL-1β, IL-6, and TNF-α, while restoring levels of superoxide dismutase, catalase and glutathione, and decreasing monoester diterpene alkaloid levels in the kidney by inhibiting NF-κB pathway and increasing Nrf2/HO-1 pathway 196. Treatment with loganetin ameliorated rhabdomyolysis-induced AKI by inhibiting TLR4 activity and suppressing NF-ƙB and c-Jun N-terminal kinase/p38 pathways 197. Natural polysaccharides, important bioactive components derived from traditional Chinese medicine, are recognized for their therapeutic potential in treating various diseases by improving gut microbial dysbiosis 198-200. The latest study revealed that alginate oligosaccharide inhibited renal I/R injury by upregulating mannose receptor type C1 expression and suppressing TLR4/myeloid differentiation primary response 88/NF-ƙB/IL-1β inflammatory pathway 194. Moreover, selenium-enriched crude polysaccharide (SeRRP) ameliorated cadmium-induced AKI by elevating relative abundance of Muribaculaceae, Ruminococcaceae, and Lachnospiraceae 201. In addition, treatment with 5,2'-dibromo-2,4',5'-trihydroxydiphenylmethanone alleviated acute pyelonephritis by inhibiting inflammatory mediators and regulating immune responses 202 (Table 1). Therefore, these findings demonstrate the potential for natural products to affect gut-kidney axis, thereby mitigating AKI positively.

#### 4.2.2 Regulating gut microbial-derived metabolites in CKD by natural products

Compared with AKI, numerous research has reported that natural products improved CKD and inhibited renal fibrosis 203-209. Increasing evidence has shown that traditional Chinese medicines ameliorated patients with CKD by reshaping microbial dysbiosis 210-212. Accumulated studies have demonstrated that natural products can ameliorate renal injury in CKD patients 213-215. For instance, a randomized controlled trial demonstrated that Yi-Shen-Hua-Shi enriched SCFA-producing bacteria and reduced uremic toxin-producing bacteria in CKD patients, resulting in decreased proteinuria and improved renal function 210. Zicuiyin decoction increased relative abundance of Prevotellaceae and Lactobacillaceae and decreased Clostridiaceae, Enterobacteriales, and Micrococcaceae in DKD patients 216. Jin Gui Ren Qi Pill and western medicine enhanced the abundance of *Prevotella_7* within the gut microbiota of DKD patients, exhibiting superior efficacy in alleviating clinical symptoms compared to western medicine treatment alone 217. Modified Zexie decoction could enhance microbial diversity, increase SCFAs, alleviate inflammation, improve renal function, and alleviate phlegm-dampness hypertension in patients by regulating the gut-immune-kidney axis 218. It was ascertained that Shen-Shuai-Ning granule could reduce the total serum IS concentration in PD patients at weeks 4, 8, and 12. Nevertheless, further large-scale, long-term clinical trials were warranted for validation 219. Fushen granule showed beneficial therapeutic effects in PD patients by increasing relative abundance of *Rothia*, *Megamonas*, and *Bacteroides*, which is closely associated with improved amino acid metabolism (Table 2) 212.

Accumulating evidence has demonstrated that natural products play a crucial role in preserving intestinal barrier integrity and regulating microbial dysbiosis in CKD. For instance, fisetin protected against hyperuricemia-induced CKD by modulating gut microbiota-derived tryptophan metabolites and activating AHR signalling pathway 220. Moreover, berberine inhibited tyrosine metabolism within gut microbiota dysbiosis and reduced nephrotoxic TMAO levels 221. Previous studies have revealed that a number of natural components mitigated DKD by increasing SCFA levels. For example, punicalagin increased SCFA concentrations and inhibited serum diamine oxidase and LPS levels, possibly alleviating renal injury through gut-kidney axis 222. Rutin, lobetyolin and luteolin, the primary active components of the Hong Guo Ginseng Guo, mitigated DKD by regulating gut microbiota and inhibiting NLRP3 inflammasome 223. A graminan type fructan from *Achyranthes bidentata* alleviated renal injury in DKD mice by restoring barrier function, modulating microbial dysbiosis, and increasing SCFAs levels 224. In addition, flavonoids derived from *Opuntia ficus-indica* fruit attenuated renal injury in DKD mice by altering gut microbiota, suppressing LPS-induced inflammation, and promoting SCFA production 225. In hyperuricemic rats, *Polygonum cuspidatum* rectified amino acid metabolic disorders including those of tyrosine, leucine, and phenylalanine by modulating gut microbiota dysbiosis 226. Jinlida reshaped the gut microbiota, elevated pyridoxamine and pyridoxine levels, inhibited the advanced glycation end-products (AGEs)-the receptor for AGEs (RAGE) axis, and thereby suppressed downstream Smad2-mediated fibrosis and NF-κB-driven inflammation, exerting a renoprotective effect 227. Guizhi Fuling pills might ameliorate renal injury in mice by regulating the NLRP3 inflammasome, Nrf2 pathway, and gut microbial composition 228. Simiao decoction alleviated hyperuricemia-induced renal injury by modulating gut microbial metabolism of tyrosine and tryptophan, thereby decreasing uremic toxins such as IS and PCS 229. Polysaccharides derived from natural products have a broad spectrum of sources and possess various pharmacological effects 230, 231. Fucoidan improved renal fibrosis by *Akkermansia muciniphila*-produced acetate, which subsequently inhibited neuraminidase-1/TLR4/NF-κB pathway 232. In addition, *Dioscorea septemloba* polysaccharides alleviate renal disease and hyperuricemia by improving intestinal barrier function injury, gut microbial dysbiosis and lipid metabolism disorder (Table 2) 233. Rhamnogalacturonan I from *Polygonum aviculare* L. regulated the gut microbiota and promoted taurine production, thereby inhibiting TNF-α expression, relieving local inflammation, and exerting anti-nephrolithiatic effect 234. Taken together, natural products are a potentially therapeutic approach for treating kidney disease.

### 4.3 Regulating gut microbial-derived metabolites in CKD by microecological therapy

A growing body of evidence shows that microbial therapeutics including probiotics, prebiotics, and synbiotics 35, 154, 235 are the most commonly used method for regulating gut microbiota in diverse diseases 236, 237. Supplementation with *Faecalibacterium prausnitzii* attenuated renal inflammation, renal insufficiency, and decreased serum levels of numerous uremic toxins in CKD mice, which was partially attributed to butyrate-mediated renal GPR-43 signaling 111. Supplementing with probiotic *Bifidobacterium animalis* reduced relative abundance of *Fusobacterium nucleatum* and *Eggerthella lenta*, decreased uremic toxin levels and slowed the progression of kidney disease in CKD rats 40. Supplementation with the probiotic DM02, comprising *L. plantarum* and *L. reuteri*, reduced IS and* E. coli* levels in patients with stage 3-5 CKD, attenuated heart failure progression in CKD rats, and lowered levels of nicotinic acid, IS, and N-acetylserotonin 238. In 5/6 nephrectomy mice, *Lactobacillus rhamnosus* L34 alleviated uremia-induced systemic inflammation by decreasing uremic toxins such as IS and TMAO and gut leakage, thereby delaying CKD progression 239. Additionally, supplementation with β-glucan prebiotic in CKD patients with stage 3 to 5 reduced colon-derived uremic toxins PCS, IS, and p-cresyl glucuronide levels, and showed a trend toward shifting the gut microbiota from a *Bacteroides 2* to *Prevotella* enterotype 240. Administering a prebiotic mixture of oligofructose and inulin alleviated renal function and frailty among the older population, while increasing the levels of alanine, histidine, and methionine. Within the microbiota, *Dialister* emerged as a key species, exhibiting positive correlations with indole-3-lactic acid, L-tryptophan, indole, and L-alanine 241. Adenine-induced CKD rats supplemented with prebiotic gum acacia exhibited reduced Tenericutes, Verrucomicrobia, Proteobacteria, and Actinobacteria abundance, slowing down CKD progression by enhancing butyrate production 242. The combination of *Lactiplantibacillus plantarum* MPB-65 and epicatechin as a synbiotic supplement mitigated hyperuricaemia by regulating the gut-kidney axis. It increased SCFAs levels, reduced serum BUN, uric acid, and creatinine levels, and inhibited hepatic xanthine oxidase activity 243.

Despite the promising benefits, the application of microecological therapies in CKD patients also carries potential risks and side effects that warrant careful consideration. Adverse events were reported infrequently and were mild in nature in patients with stage 1 to 5 CKD who were treated with synbiotics, prebiotics, or probiotics, and included abdominal pain, diarrhoea, flatulence, and nausea 244. However, a meta-analysis of critically ill adult patients showed that probiotic and synbiotic administration was associated with an increased risk of adverse events, primarily bacteremia and intestinal ischemia 245.

In summary, although probiotics, prebiotics, and synbiotics show promise as additional therapies for kidney disease, their use should be determined following careful consideration of the patient's immune status, disease stage, and any other medications they are taking.

### 4.4 Regulating gut microbial-derived metabolites in CKD by dietary interventions

Compared to a low-fat diet rich in complex carbohydrates, long-term consumption of a Mediterranean diet enriched with extra-virgin olive oil protected renal function and slowed eGFR progression in DKD patients 246. For instance, oat dietary fiber enhanced SCFAs levels in CKD mice, rectify intestinal dysbiosis, diminished uremic toxins, and alleviated renal fibrosis through NF-ƙB/TGF-β signaling pathway 247. In summary, these strategies represent great promise as therapeutic management options for kidney disease.

## 5. Conclusion, perspective and challenges

Increasing evidence has indicated that there is a bidirectional relationship between kidney and gut microbiota. Maintaining homeostasis between the gut microbiota and the host is crucial for preventing renal fibrosis. The function and composition of gut microbiota are influenced by the uremic milieu. At the same time, an increase in microinflammation and uremic toxins derived from gut microbiota is linked to ecological disruption, both of which contribute to the progression of renal disease. Microbial-derived metabolites, including SCFAs and uremic toxins, modulate immune cells, control inflammatory mediators, and mediate the gut-mucosal barrier. Ultimately, they shape the overall environment, either disrupting or promoting the process of renal fibrosis. Using dialysis (hemodialysis, peritoneal dialysis and colonic dialysis), KT, natural product interventions, microecological therapies (probiotics, prebiotics and synbiotics), and dietary interventions to target the gut microbiota is a potential therapy for kidney disease, but it still faces several new challenges. Further research is required to clarify the pharmacological effects of significantly modified bacterial species and their metabolites in patients with kidney disease. Furthermore, it is vital to grasp the underlying molecular mechanisms of microbiological dysbiosis associated with kidney disease, which are triggered by microbial-derived metabolites. To deepen our comprehension of the physiological functions of gut microbiota and unravel the pathophysiology and etiology of kidney disease, sequencing, bioinformatics, and untargeted metabolomics technologies must be integrated. Further research is needed to investigate the underlying mechanisms of using natural products, probiotics, and prebiotics to restore homeostasis to the gut-kidney axis as a potential therapeutic strategy and to examine its clinical application. Although significant progress has been made in current research, the field still faces many challenges and opportunities. Notably, the deep integration of metagenomics with other emerging technological fields is providing unprecedented insights into the gut microbiota-kidney axis. Multi-omics integration strategies, which combine metagenomics, metatranscriptomics, metaproteomics, and metabolomics, enable the systematic analysis of interactions between the gut microbiota and the host at multiple levels, including composition, function, and metabolites. They also allow the systematic analysis of microbial functional and metabolite networks. Single-cell sequencing and spatial transcriptomics reveal heterogeneous responses of renal and intestinal cells to microbial metabolites. Furthermore, engineered strategies such as gene-edited probiotics, synthetic microbiota and phage therapy provide new opportunities for the precise regulation of harmful metabolites. However, the clinical translation of these technologies is hindered by challenges such as insufficient standardisation, high costs, poor model interpretability and difficulties in validating causal relationships. In the future, large-scale, multicentre, longitudinal follow-up cohorts should be established to integrate multi-omics and clinical data. This will facilitate progress from association to causation and from understanding mechanisms to translating them into practice. It will also enable precise, microbiome-targeted interventions.

## Figures and Tables

**Table 1:** Treatment of natural products in patients and animal models with AKI.

Drugs	Disease	Dose	Microbial dysbiosis	Molecular mechanism	Refs
ACA	Cisplatin-induced AKI mice	5, 10 mg/kg	Increased *Bacteroides* and *Ligilactobacillus* abundance while decreasing *Clostridium* abundance.	Restored glutathione and tryptophan metabolism and improved IƙB/NF-ƙB and Keap1/Nrf2 pathways.	196
Loganetin	AKI mice	2, 18 mg/kg	-	Inhibited TLR4 activity and suppressed NF-ƙB and JNK/p38 pathways.	197
Alginate oligosaccharide	I/R-induced AKI rats	100 mg/kg	-	Inhibited TLR4/MyD88/NF-ƙB/IL-1β pathway.	194
SeRRP	Cadmium-induced AKI mice	100 mg/kg	Increased Muribaculaceae, Ruminococcaceae and Lachnospiraceae abundance.	Modulated gut microbiota and antioxidant activity.	201

**Abbreviations:** JNK, c-Jun N-terminal kinase; Keap1, kelch-like ECH-associated protein 1; MyD88, myeloid differentiation primary response 88.

**Table 2:** Treatment of natural products including traditional Chinese medicine in patients and animal models with CKD.

Drugs	Disease	Dose	Microbial dysbiosis	Metabolites or effects	Mechanism	Refs
Yi-Shen-Hua-Shi	CKD patients	1 bag/ 3 times/day	Increased *Lachnoclostridium*, *Lachnospiraceae* and *Sutterella* abundance while decreased *Clostridium innocuum* and *Eggerthella* abundance.	Reduced 24-h proteinuria level.	Modulated gut microbiota and regulated vitamin, lipid and glycan metabolism.	210
Zicuiyin Decoction	DKD patients	150 mL/day	Increased Lactobacillaceae abundance while decreased Clostridiaceae abundance.	Decreased serum creatinine level.	-	211
Jin Gui Ren Qi Pill	DKD patients	5 g/ 2 times/day	Increased* Prevotella_7* abundance.	Reduced IL-2 level.	Modulated gut microbiota.	217
Modified Zexie decoction	Hypertension patients	150 mL/2 times/day	Increased *Streptococcus* and *Prevotella* abundance while decreased *Escherichia* and* Agathobaculum* abundance.	Increased acetate, propionate and butyrate levels.	Regulated gut-immune-kidney axis and enhancing microbial diversity.	218
Shen-Shuai-Ning granule	PD patients	5 g/3 times/day	-	Reduced IS level.	Decreased uremic toxins.	219
Fushen Granule	PD patients	18 g/2 times/day	Increased *Rothia*, *Megamonas* and *Bacteroides* abundance.	Reduced serum creatinine and urea levels.	Improving carbohydrate and amino acid metabolism.	212
Fisetin	Hyperuricemia-induced CKD mice	50,100 mg/kg	-	Decreased tryptophan metabolites.	Modulated tryptophan metabolism and AHR activation.	220
Berberine	Adenine-induced CKD rats	100 mg/kg	Increased butyric acid-producing bacteria abundance.	Increased butyric acid level while reduced TMAO level.	Inhibited tyrosine metabolism.	221
Punicalagin	High-fat diet-induced DKD mice	50, 100 mg/kg	Increased Lachnospiraceae abundance.	Increased SCFA level while reduced LPS and DAO levels.	Improving diabetic renal injury through gut-kidney axis.	222
Lobetyolin, luteolin, and rutin	Streptozotocin-induced DKD rats	75 mg/kg	Increased *Ruminococcus* and *Barnesiella* abundance while decreased *Oscillospira* abundance.	Increased SCFA level while reduced LPS level.	Modulated gut microbiota.	223
ABPW1	Streptozotocin-induced DKD mice	300 mg/kg	Increased *Bacteroide* abundance while decreased *Alistipes*, *Faecalibaculum*, *Rikenella*, and *Laedolimicola* abundance.	Increased SCFA level.	Modulated gut microbiota.	224
OFI-F	Streptozotocin-induced DKD mice	300, 500 mg/kg	Increased *Akkermansia* and *Bacteroides* abundance while decreased *Alistipes*, *Helicobacter*, and *Desulfovibrio* abundance.	Increased SCFA level.	Regulated gut microbiota and promoting SCFAs production.	225
*Polygonum cuspidatum*	Hyperuricemia patients, potassium oxonate and adenine-induced rats	2, 20, 200 mg/kg	Increased *Lactobacillus* and *Candidatus_Arthromitus* abundance while decreased *Pseudomonas*, *Helicobacter*, and *Romboutsia* abundance.	Decreased leucine, phenylalanine, and tyrosine levels.	Rectifying amino acid metabolism disorders by modulated gut microbiota dysbiosis.	226
Jinlida	DKD mice	1.75, 3.5 mg/kg	Increased *Paramuribaculum intestinale* abundance.	Increased pyridoxamine and pyridoxine levels.	Reshaping gut microbiota and suppressed AGEs-RAGE axis.	227
Guizhi Fuling pills	Carbon tetrachloride-induced mice	125 mg/kg	Increased *Bacteroidales-S24-7-group* abundance while decreased *Lactobacillus* abundance.	Decreased IL-6, IL-1β, and TNF-6 levels.	Regulated NLRP3 inflammasome, Nrf2 pathway, and gut microbial composition.	228
Simiao decoction	Uricase-knockout rats	3, 6 g/L drugs in drinking water	Increased *Lactobacillus_intestinalis* abundance.	Decreased IL-6, IL-1β and MCP-1 levels and downregulated uremic toxins including IS, PCS, and methylguanidine.	Modulated gut dysbiosis and decreased uremic toxins.	229
Fucoidan	UUO-induced mice	25, 50, 100 mg/kg	Increased *Akkermansia muciniphila* abundance.	Increased SCFA level.	Inhibited NEU1-TLR4-NFƙB-mediated inflammation.	232
DSP	Hyperuricemia mice	100, 200, 300 mg/kg	Increased *Akkermansia* and *Lactobacillus* abundance while decreased *Bacteroides* abundance.	Reduced TNF-α, IL-1β, IL-6, and LPS levels.	Inhibited NF-ƙB pathway by gut-kidney axis.	233
RG-I	Glyoxylate-induced rats	10, 20, 40 mg/kg	Increased Enterobacterales, *Lactobacillus*, and *Mediterraneibacter* abundance.	Increased taurine level while reduced TNF-α level.	Regulated gut microbiota and suppressed TNF-α expression in kidneys.	234

**Abbreviations:** ABPW1, a graminan type fructan from *Achyranthes bidentata*; APSP, extract mixture of *Andrographis paniculata* (Burm. f.) Wall. ex Nees and *Syzygium polyanthum* (Wight.) Walp leaves; BSA, barleriside A; DAO, diamine oxidase; DSP, *Dioscorea septemloba* polysaccharides; HMF, 5,6,7,8,3',4'-hexamethoxyflavone; NEU1, neuraminidase-1; OFI-F, flavonoids derived from *Opuntia ficus-indica* fruit; RG-I, rhamnogalacturonan I from *Polygonum aviculare* L.; UUO, unilateral ureteral obstruction; 16(R)-HETE, 16(R)-hydroxy-5,8,11,14-eicosatetraenoic acid.
